# Supplementary material for: Small GTPase ActIvitY ANalyzing (SAIYAN) system: A method to detect GTPase activation in living cells
Source: J Cell Biol. 2024 Aug 5;223(10):e202403179. doi: 10.1083/jcb.202403179 (PMC11303508; doi:10.1083/jcb.202403179)
Supplement: Table S1 — shows siRNA sequences used in this study. [file JCB_202403179_TableS1.docx]

Supplementary Table 1: siRNA sequences used in this study

| oligo | sense | antisense |
| --- | --- | --- |
| cTAGE5 (1825) | CCG CCA GGA CAA UCA UAU CCU GAU U | AUC AGG AUA UGA UUG UCC UGG CGG |
| cTAGE5(2176) | GCC AUG UUU GGA GCU UCU CGA GAU U | AAU CUC GAG AAG CUC CAA ACA UGG C |
| cTAGE5 (92) | GAC CAG AUU CUA AUC UUU AUG GUU U | AAA CCA UAA AGA UUA GAA UCU GGU C |
| Sar1A (269) | UCC CAG CAA UUA AUG GGA UUG UCU U | AAG ACA AUC CCA UUA AUU GCU GGG A |
| Sar1A (237) | CGA GCA AGC ACG UCG CGU UUG GAA A | UUU CCA AAC GCG ACG UGC UUG CUC G |
| Sec12 (1214) | CCA UCC UGC UGC UCC AGA GUG CCU U | AAG GCA CUC UGG AGC AGC AGG AUG G |
| Sec12 (446) | CAG ACU UUA GCU CCG AUC CAC UGC A | UGC AGU GGA UCG GAG CUA AAG UCU G |
| Sec16A (2177) | GGG CGC AAA GUG AGC UGC CAG AUU U | AAA UCU GGC AGC UCA CUU UGC GCC C |
| Sec16A (3031) | CCG UCC CAU UCU GAC AGC CUC GCU U | AAG CGA GGC UGU CAGAAU GGG ACG G |
| Sec16A (5393) | CCC UGC CUA GUU UCC AGG UGU UUA A | UUA AAC ACC UGG AAA CUA GGC AGG G |
| Sec23A (1119) | GGG UGA UUC UUU CAA UAC UUC CUU A | UAA GGA AGU AUU GAA AGA AUC ACC C |
| Sec23A (366) | GCG UGG UCC UCA GAU GCC UUU GAU A | UAU CAA AGG CAU CUG AGG ACC ACG C |
| Sec31A (1805) | CCA UAG CAG GUG GAC AAG AAC UCU U | AAG AGU UCU UGU CCA CCU GCU AUG G |
| Sec31A (3297) | CCA GGC CAA UAA GCU GGG UGU CUA A | UUA GAC ACC CAG CUU AUU GGC CUG G |
| TANS (-32) | GAA UUG UCG CUU GCG UUC AGC UGU U | AAC AGC UGA ACG CAA GCG ACA AUU C |
| TANL (552) | CAA CUC AGA GGA AAG UGA UAG UGU A | UAC ACU AUC ACU UUC CUC UGA GUU G |
| TANS+L (4093) | CAG GAA AUC GAA GAC UGG AGU AAA U | AUU UAC UCC AGU CUU CGA UUU CCU G |
| TANS+L (4442) | CCG UGU CCA CUA AAU GUA ACC UGG A | UCC AGG UUA CAU UUA GUG GAC ACG G |
