# Supplementary material for: Small GTPase ActIvitY ANalyzing (SAIYAN) system: A method to detect GTPase activation in living cells
Source: J Cell Biol. 2024 Aug 5;223(10):e202403179. doi: 10.1083/jcb.202403179 (PMC11303508; doi:10.1083/jcb.202403179)

Fig. 2F

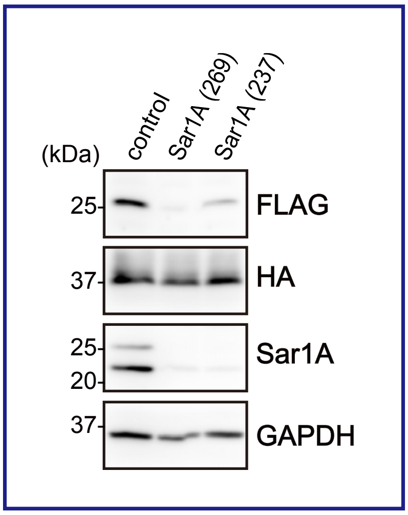

IB : FLAG

cropped data | raw tiff data | membrane

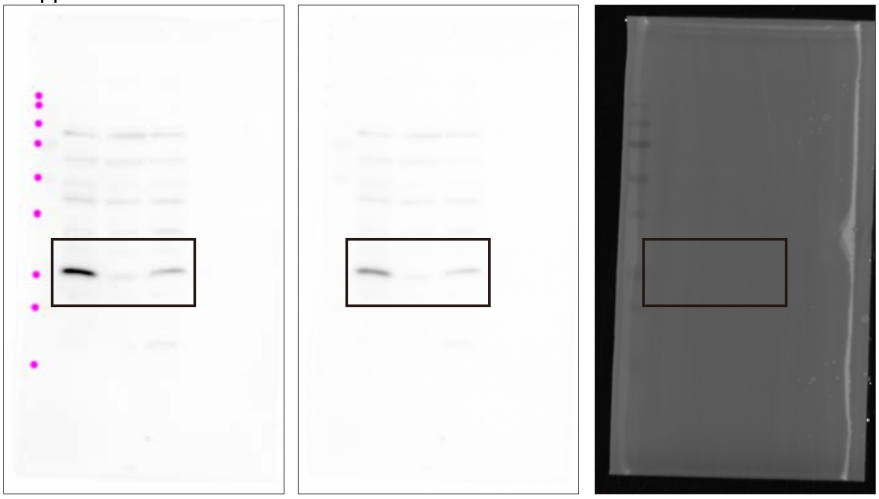

IB : HA

cropped data | raw tiff data | membrane

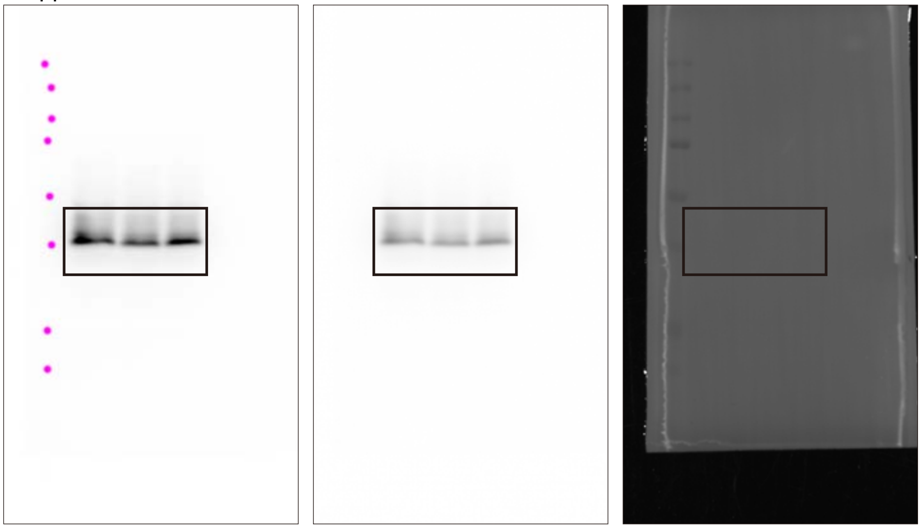

IB : Sar1A

cropped data | raw tiff data | membrane

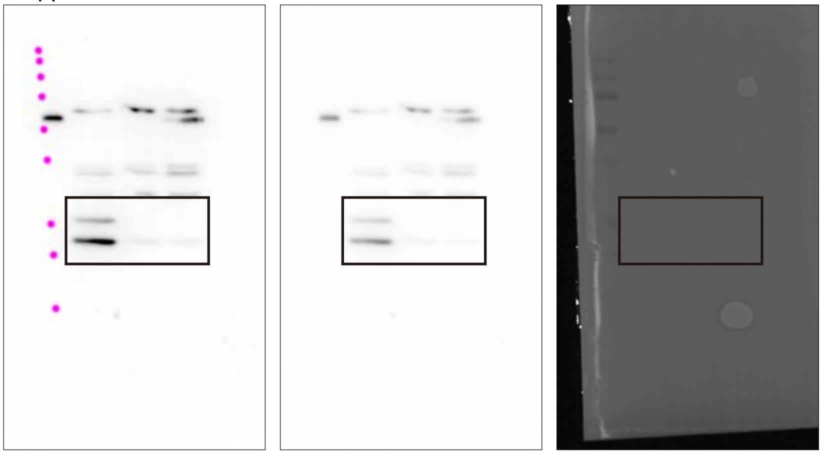

IB : GAPDH

cropped data | raw tiff data | membrane

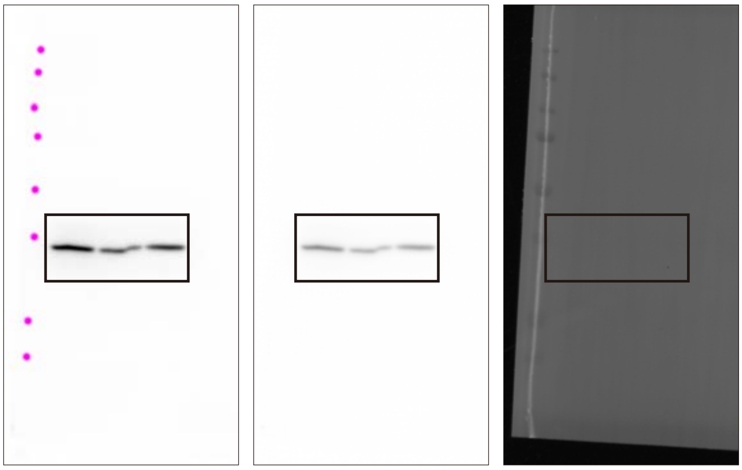

Fig. 2G

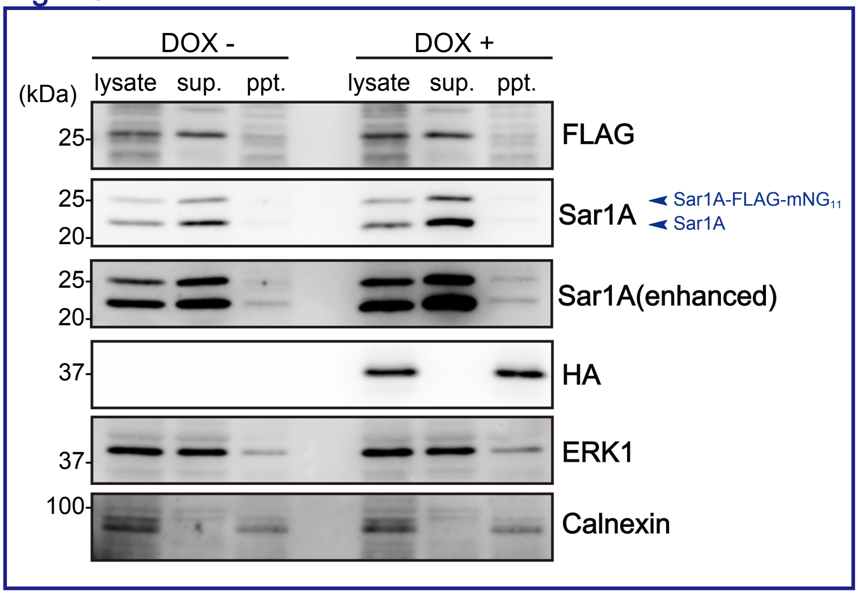

IB : FLAG

cropped data

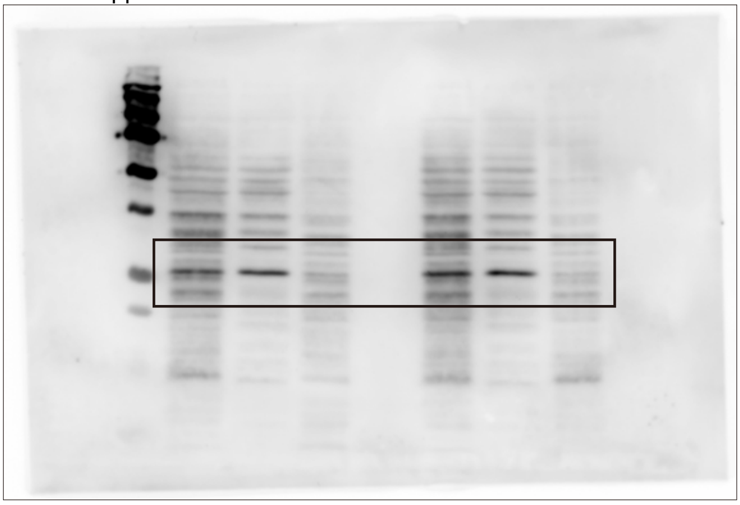

raw tiff data

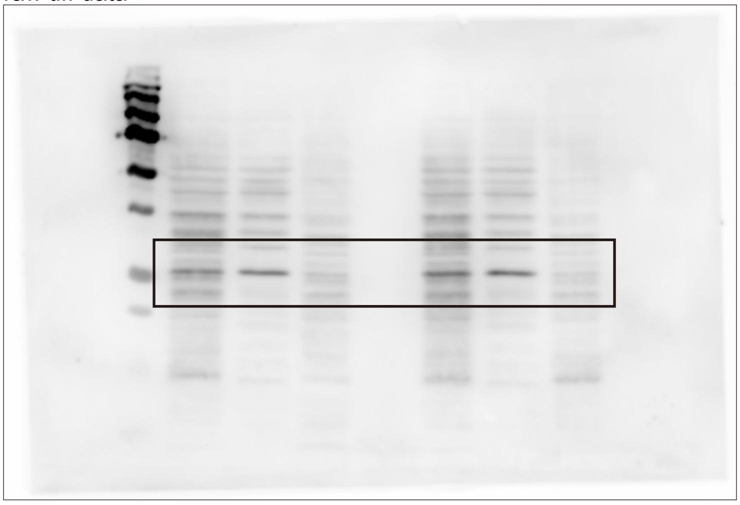

membrane

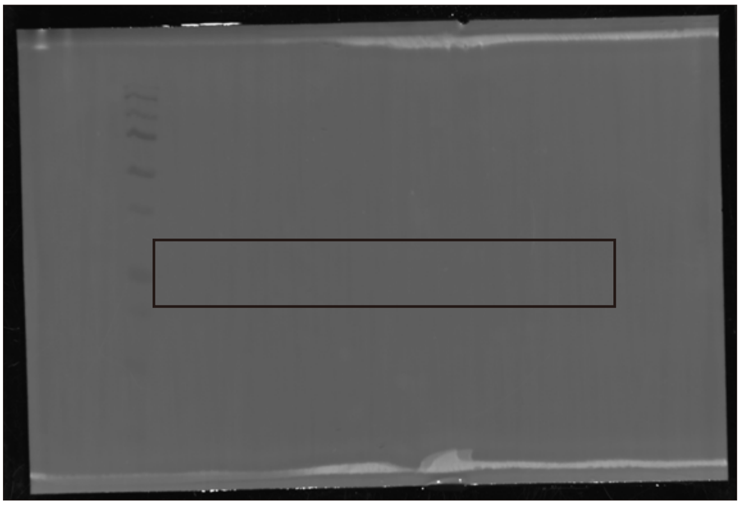

IB : Sar1A

cropped data

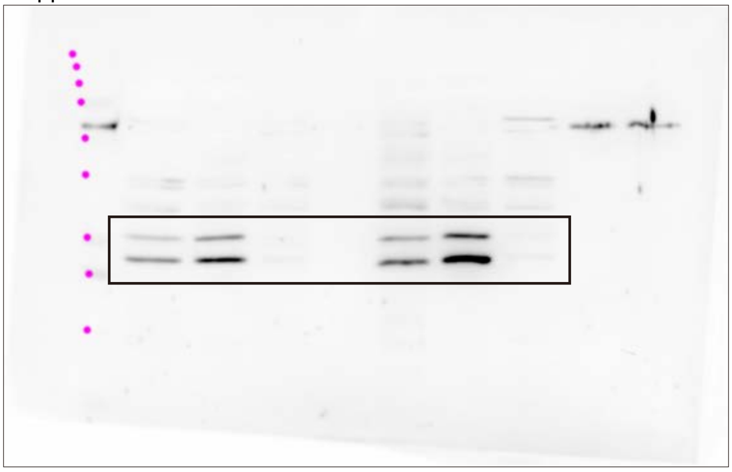

cropped data (enhanced)

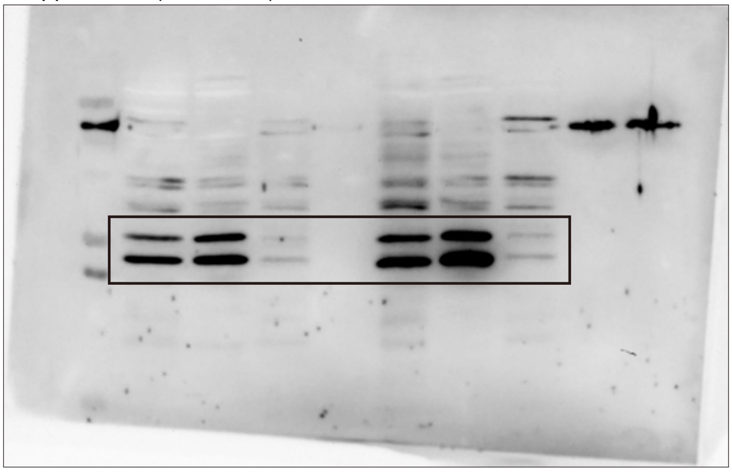

raw tiff data

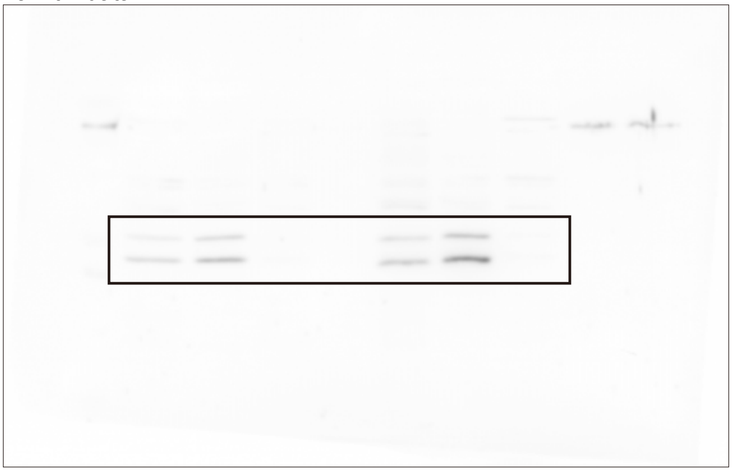

membrane

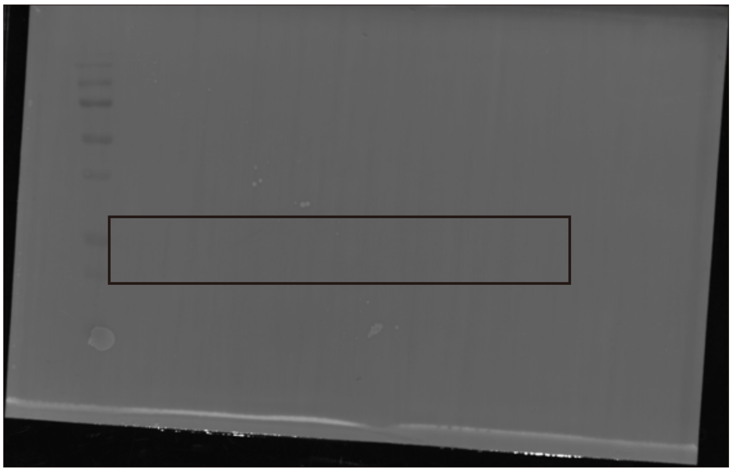

IB : HA

cropped data

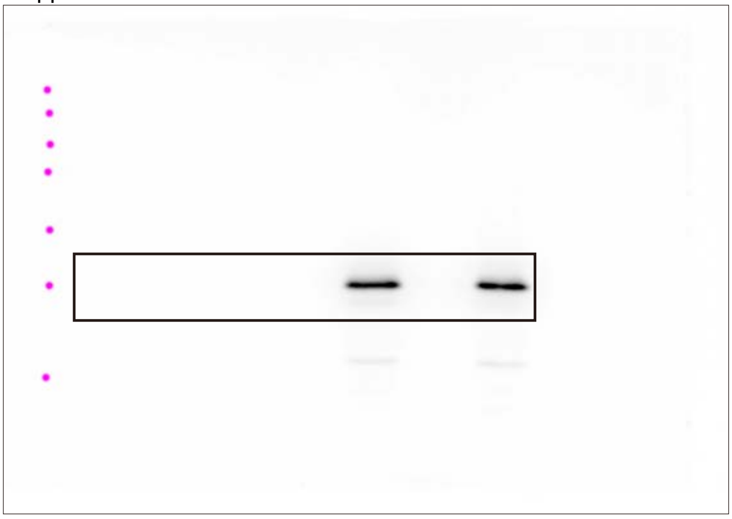

raw tiff data

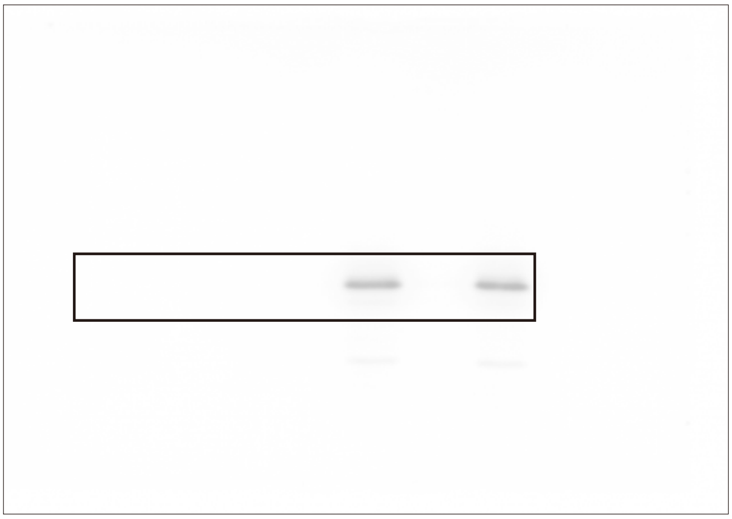

membrane

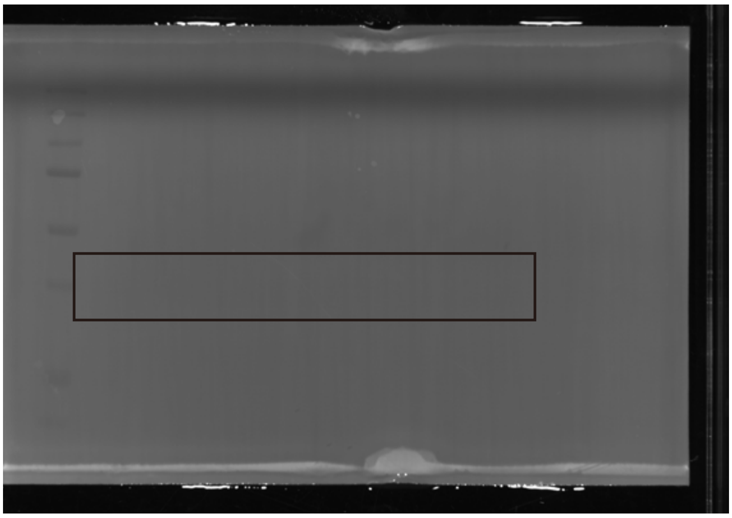

IB : ERK1

cropped data

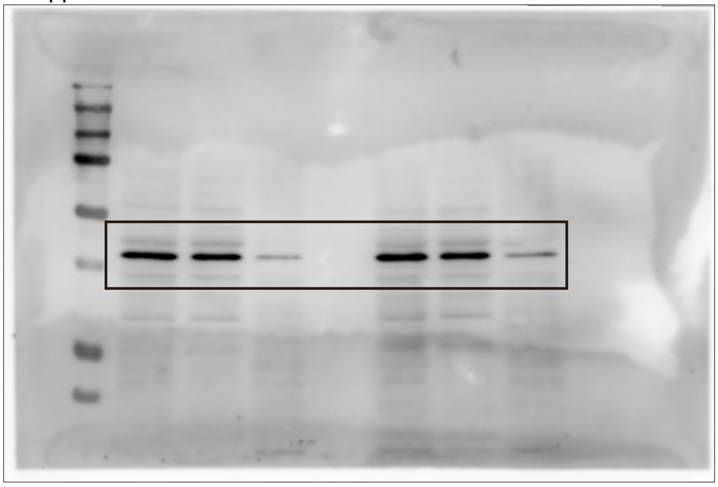

raw tiff data

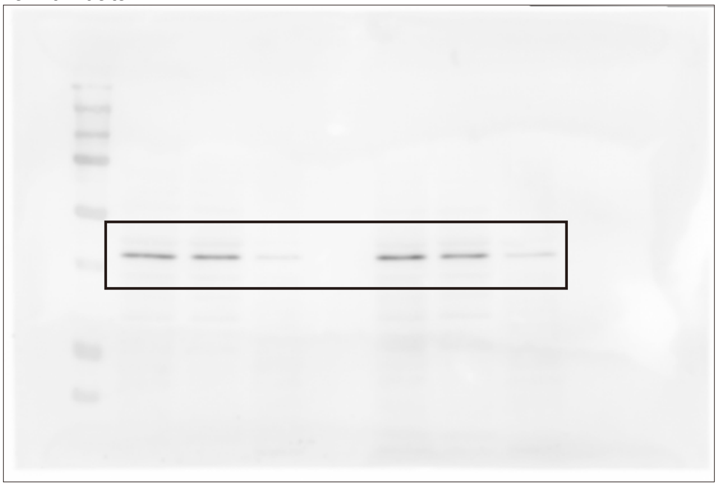

membrane

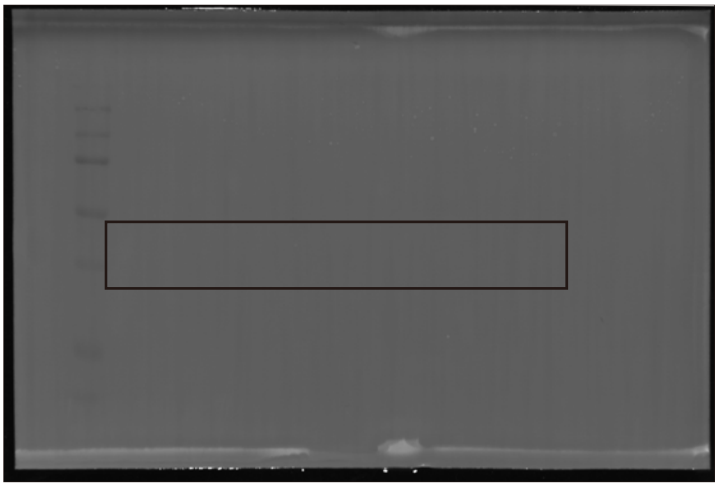

IB : Calnexin

cropped data

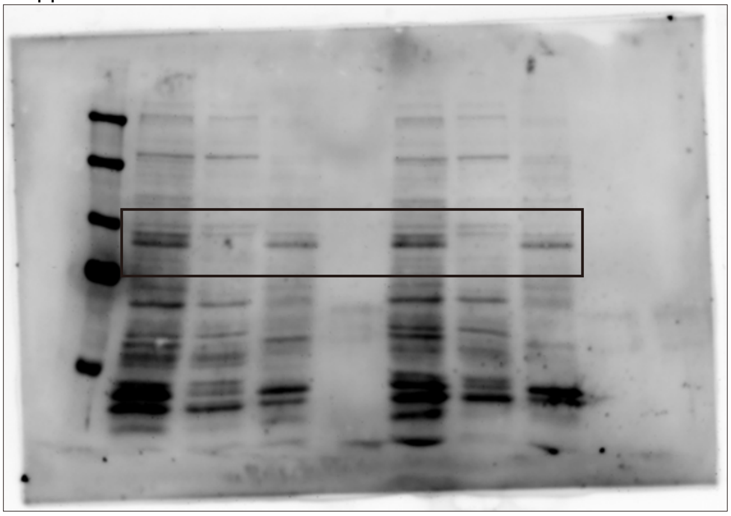

raw tiff data

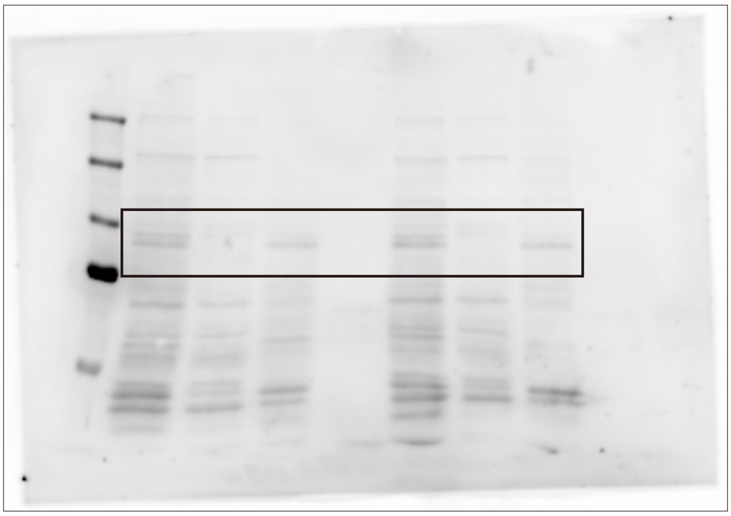

membrane

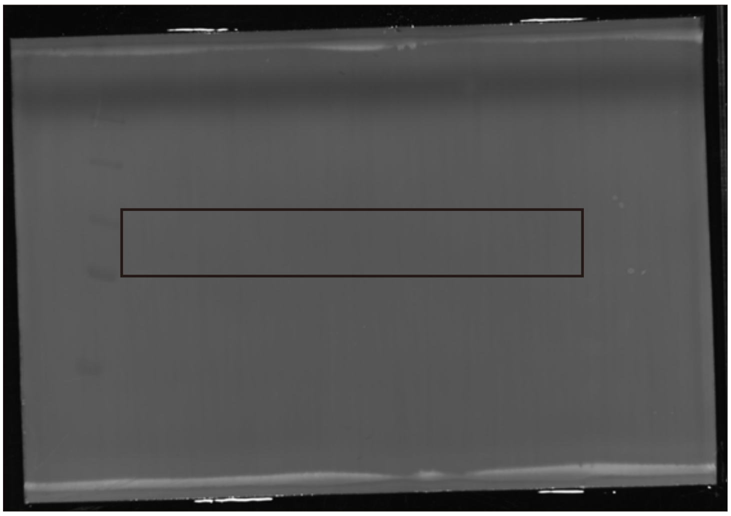

Supplement: SourceData F2 — is the source file for Fig. 2. [file JCB_202403179_SourceDataF2.pdf]
