# Supplementary material for: Small GTPase ActIvitY ANalyzing (SAIYAN) system: A method to detect GTPase activation in living cells
Source: J Cell Biol. 2024 Aug 5;223(10):e202403179. doi: 10.1083/jcb.202403179 (PMC11303508; doi:10.1083/jcb.202403179)

Fig. S3A

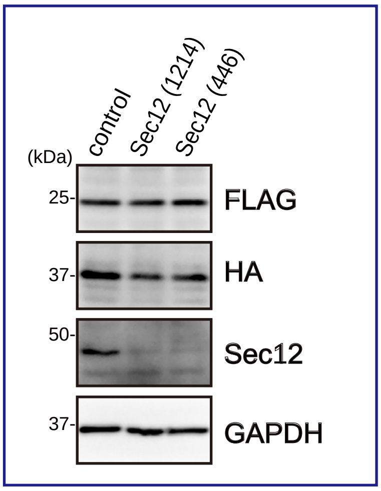

IB : FLAG

cropped data | raw tiff data | membrane

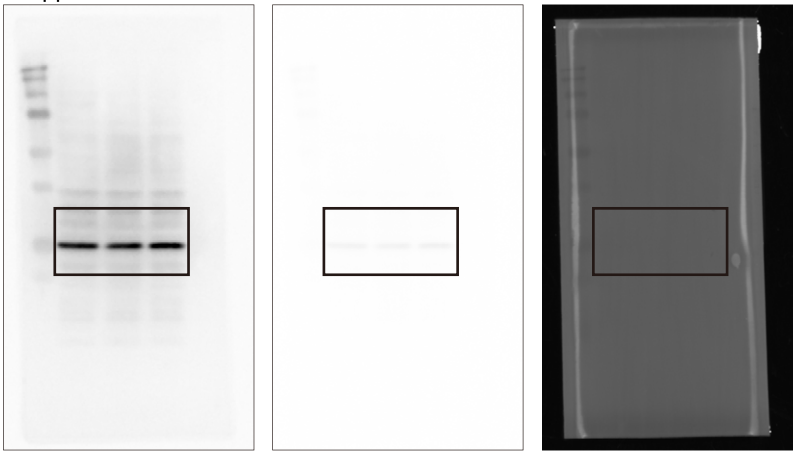

IB : HA

cropped data | raw tiff data | membrane

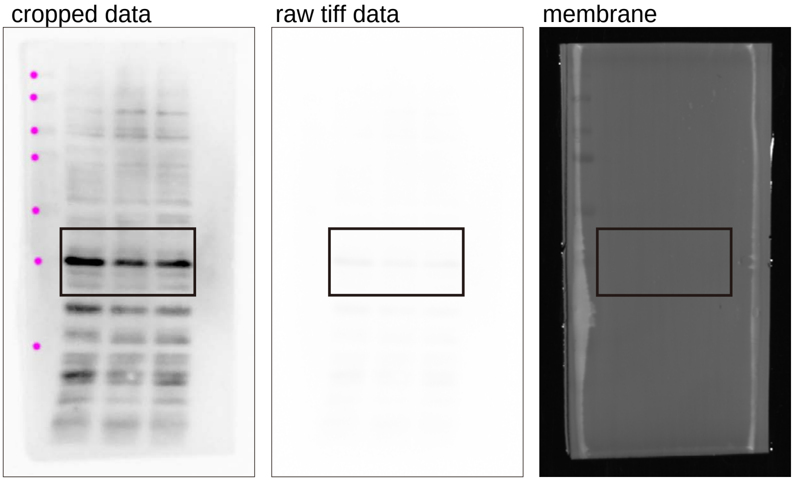

IB : Sec12

cropped data | raw tiff data | membrane

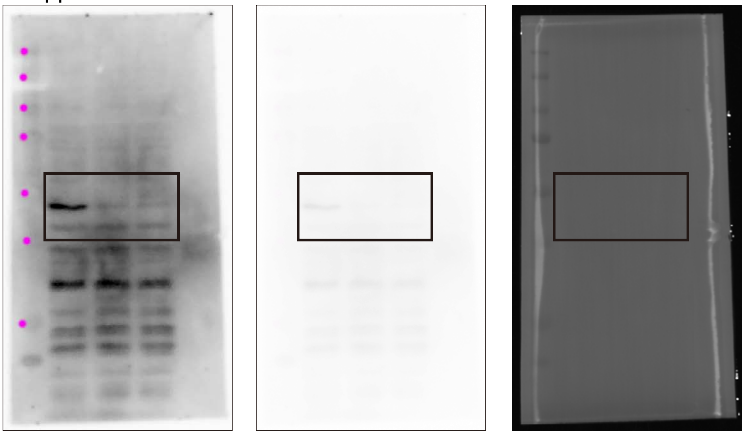

IB : GAPDH

cropped data | raw tiff data | membrane

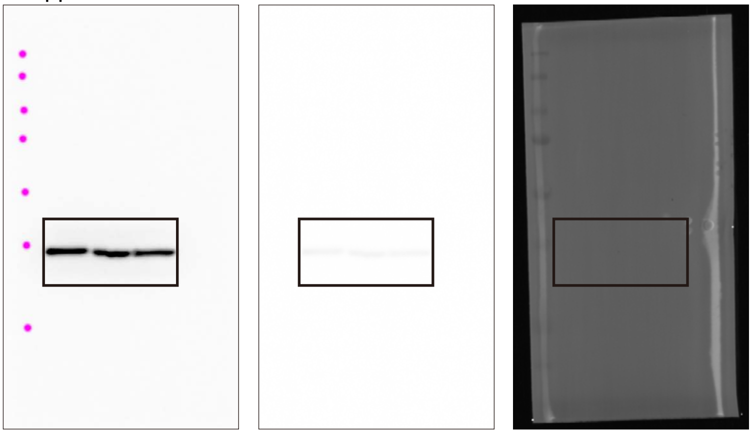

Fig. S3B

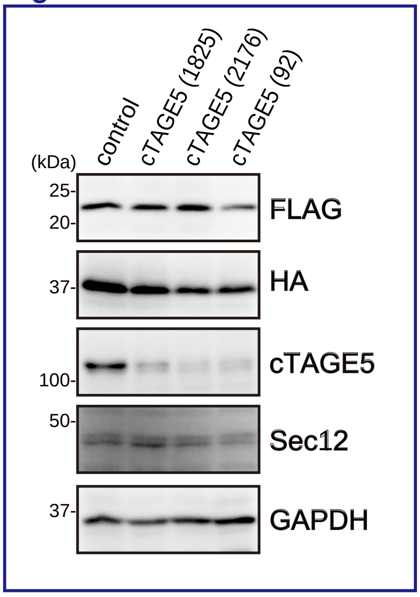

IB : FLAG

cropped data

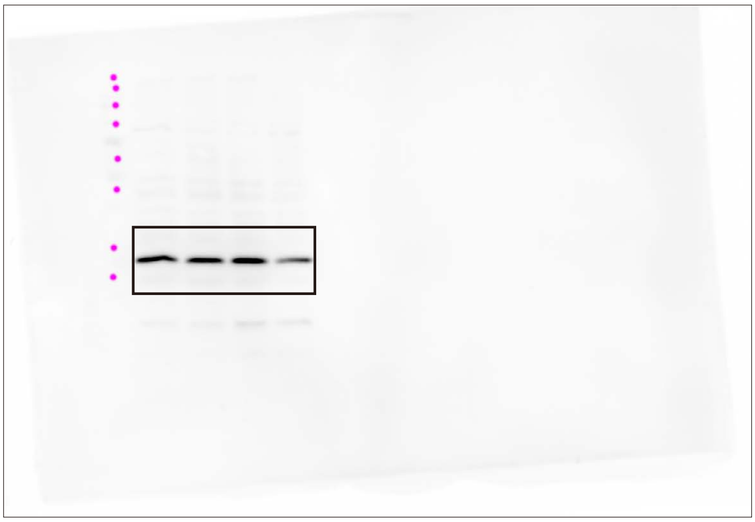

raw tiff data

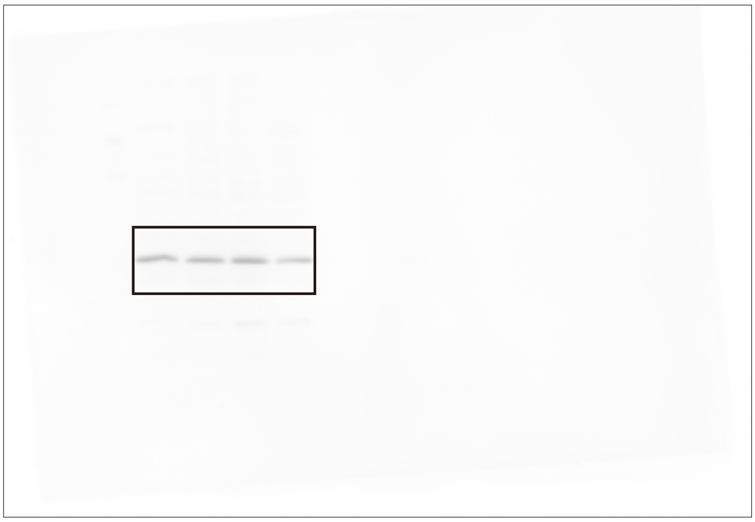

raw tiff data (enhanced)

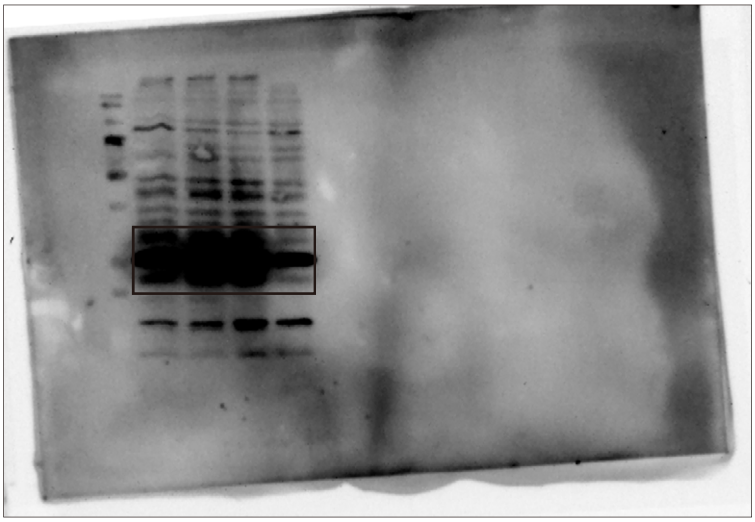

IB : HA

cropped data

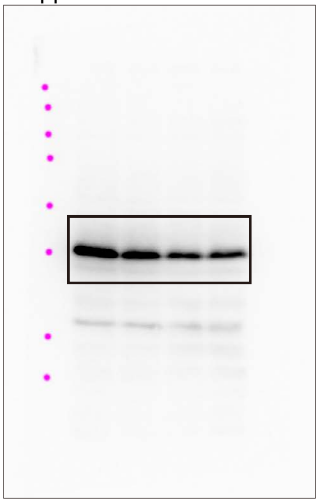

raw tiff data

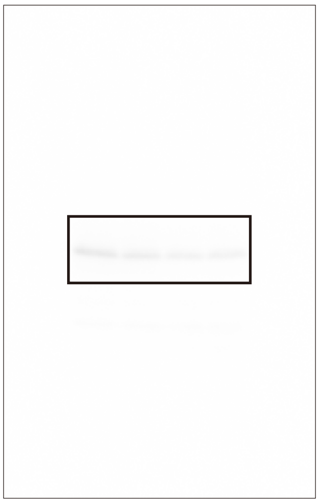

membrane

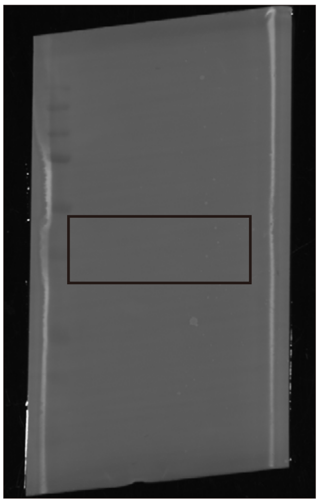

IB : cTAGE5

cropped data

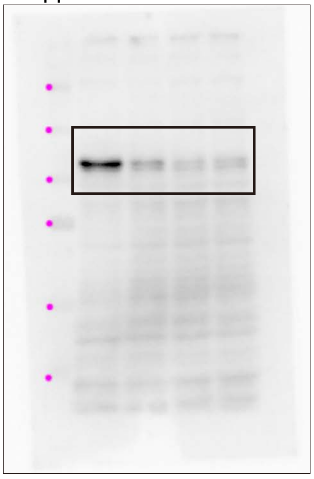

raw tiff data

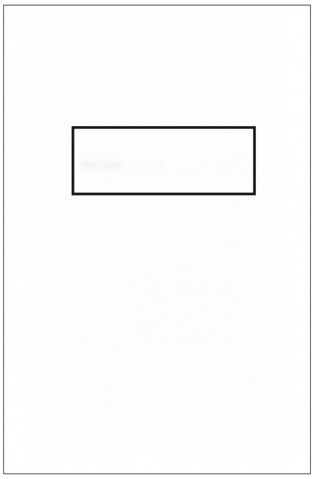

membrane

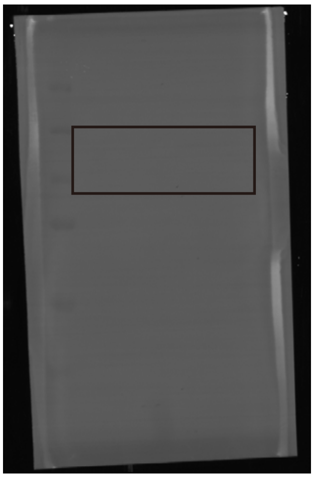

IB : Sec12

cropped data

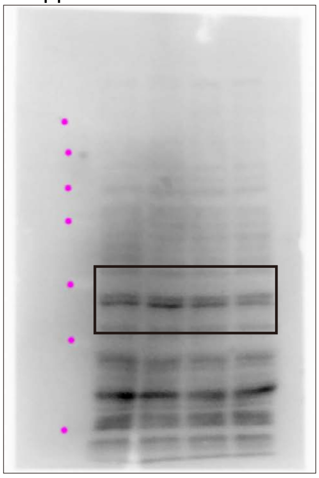

raw tiff data

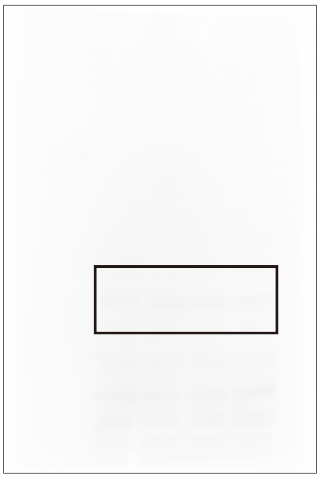

membrane

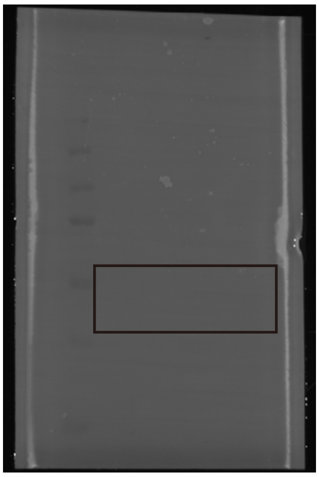

IB : GAPDH

cropped data

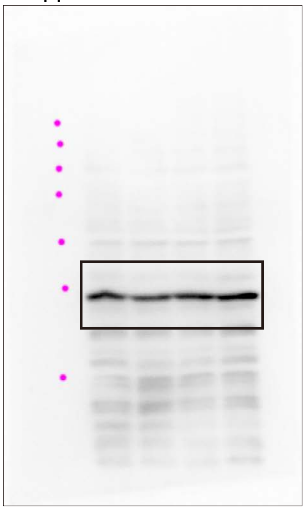

raw tiff data

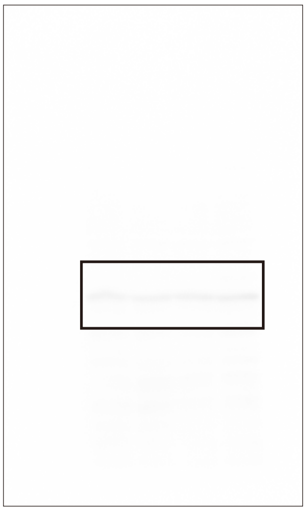

membrane

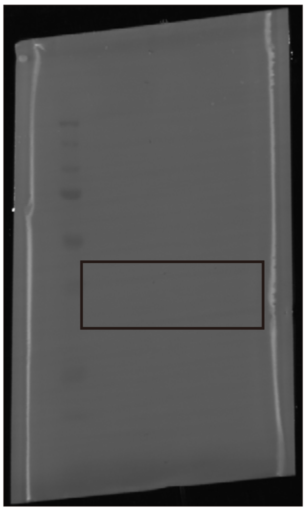

Fig. S3C

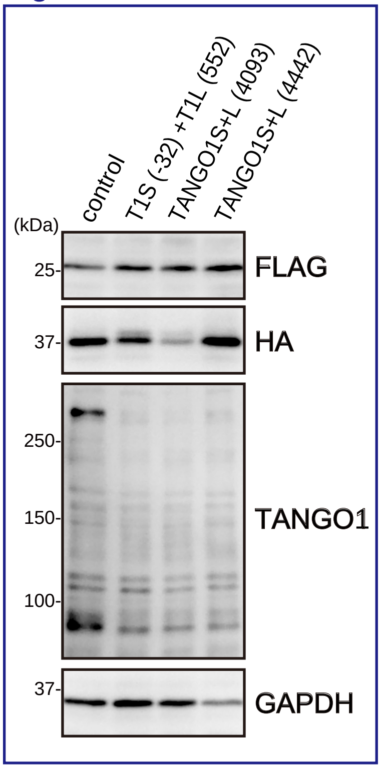

IB : FLAG

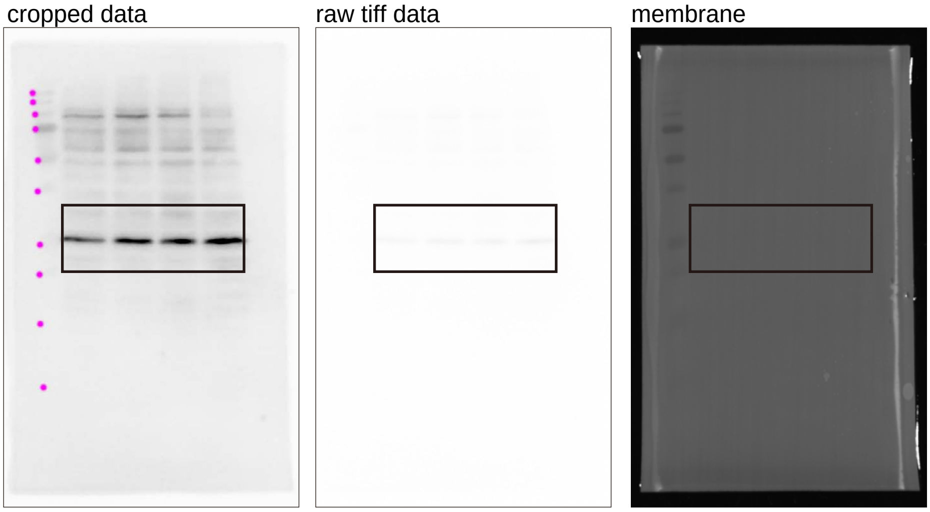

IB : HA

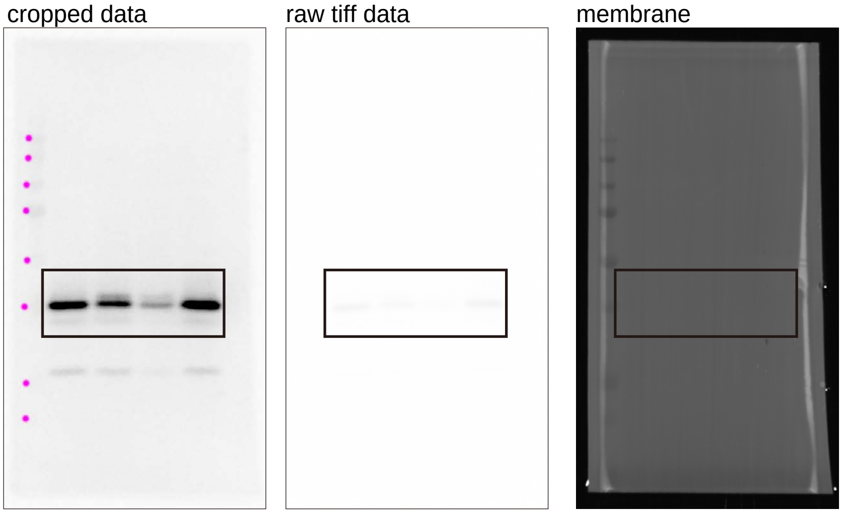

IB : TANGO1

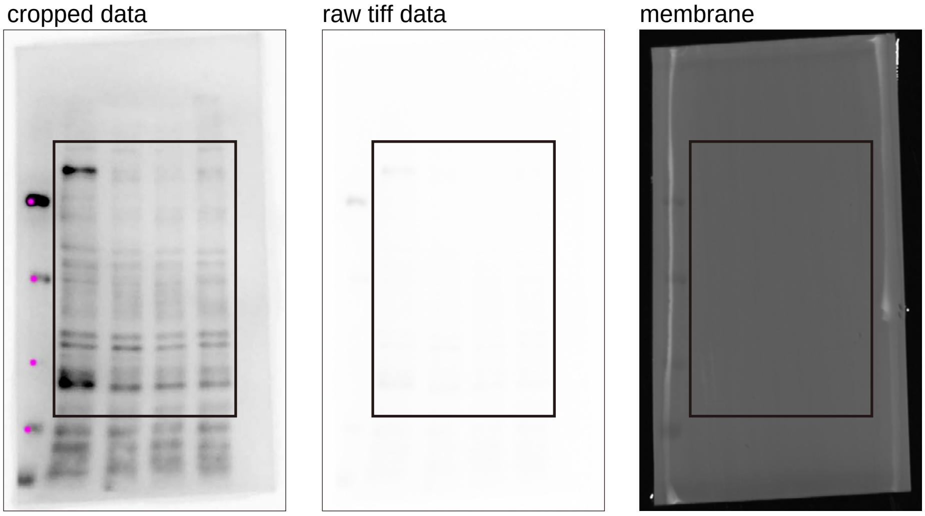

IB : GAPDH

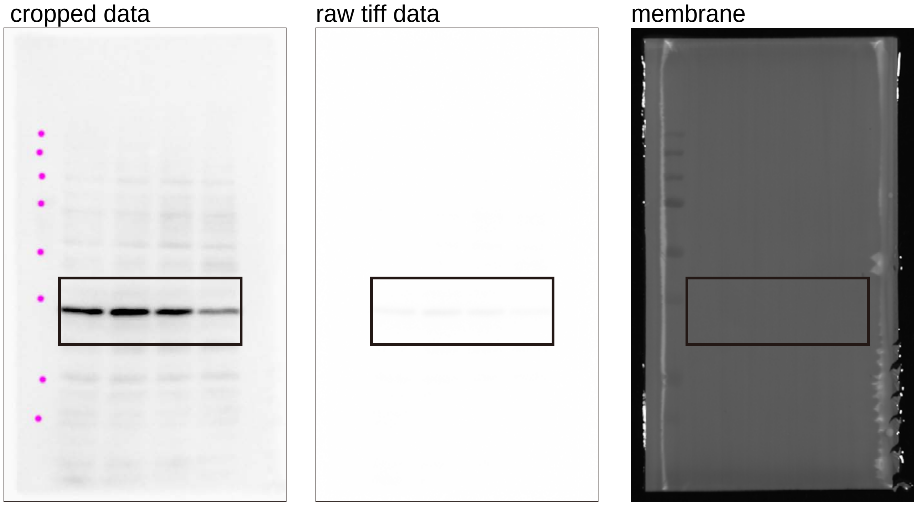

Fig. S3D

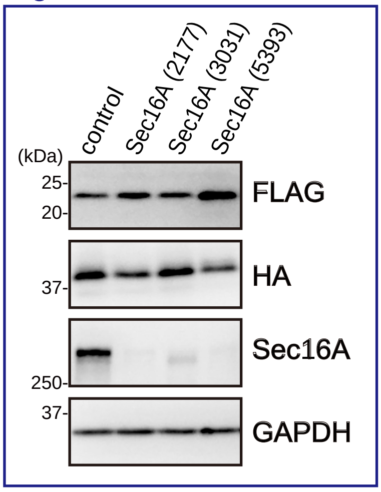

IB : FLAG

cropped data

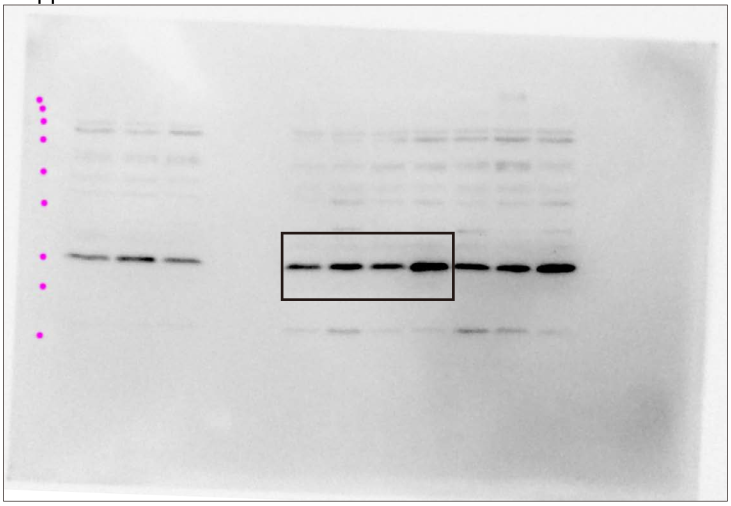

IB : HA

cropped data

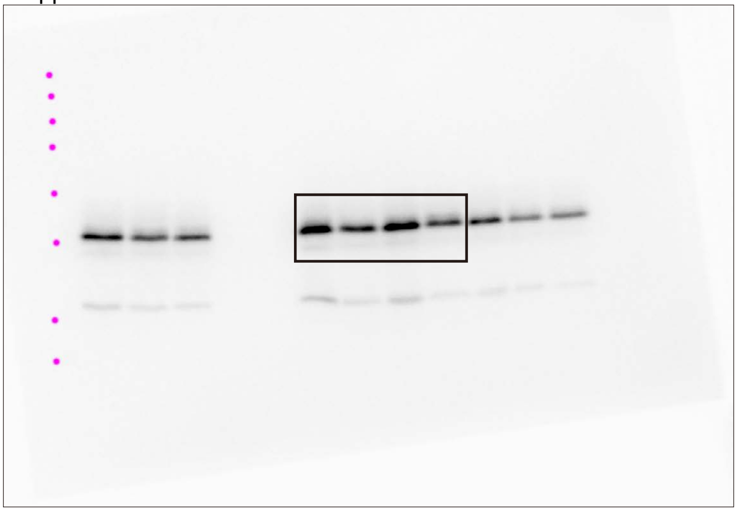

raw tiff data

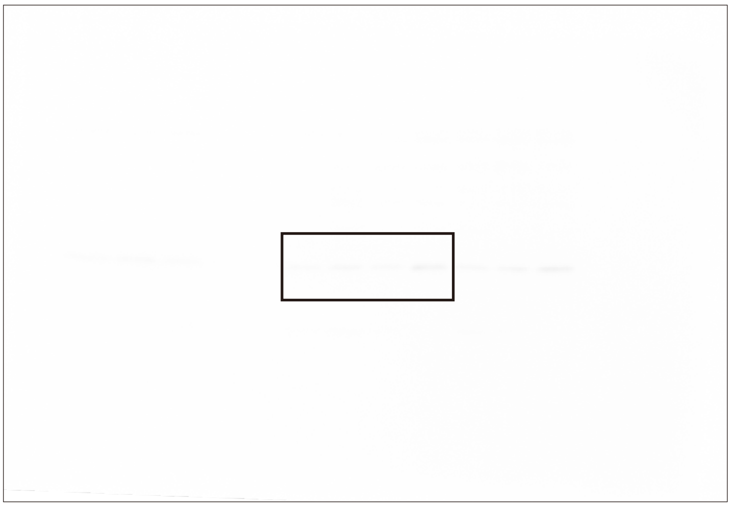

raw tiff data

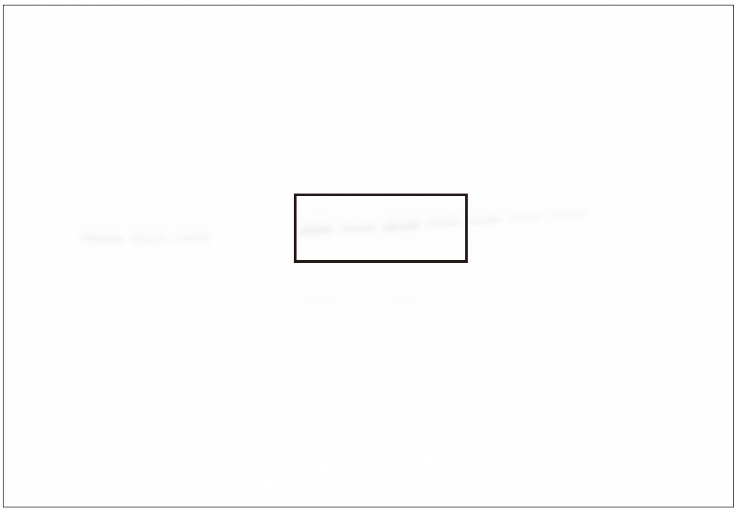

membrane

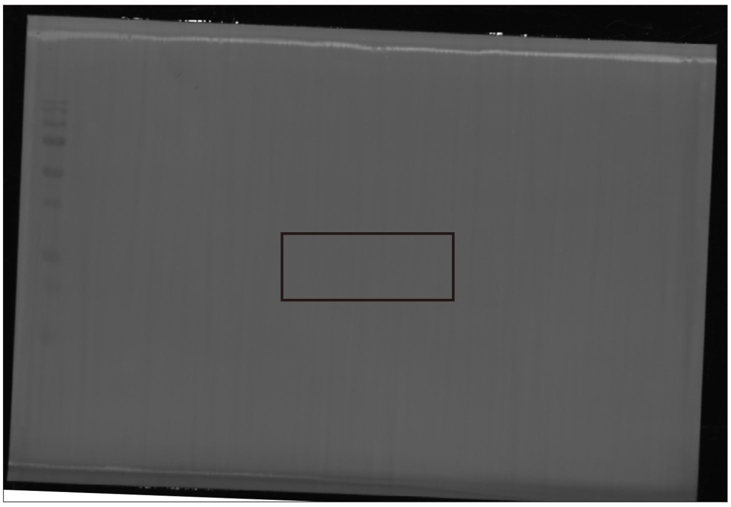

membrane

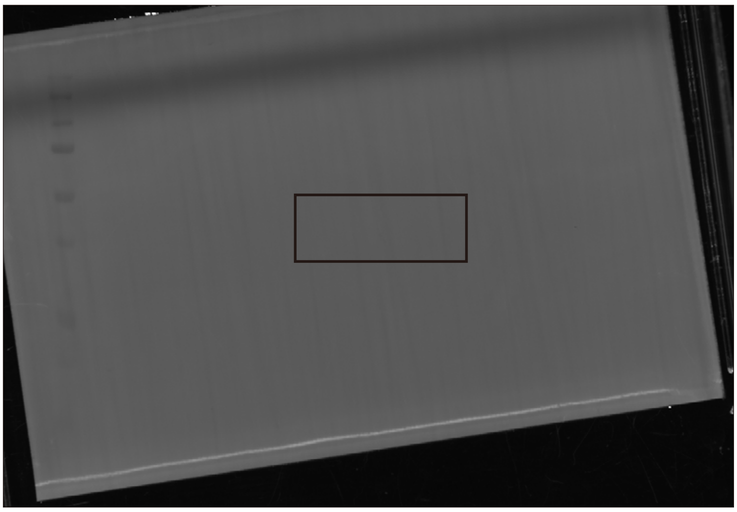

IB : Sec16A

cropped data

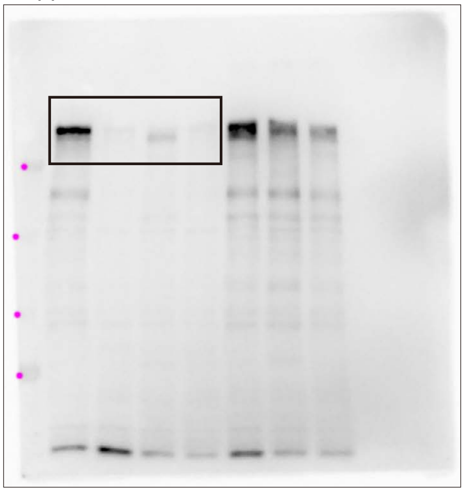

raw tiff data

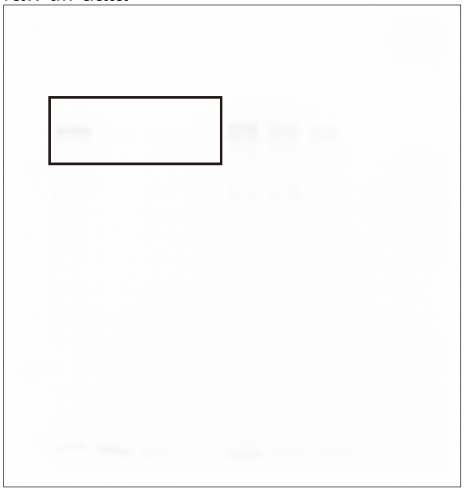

membrane

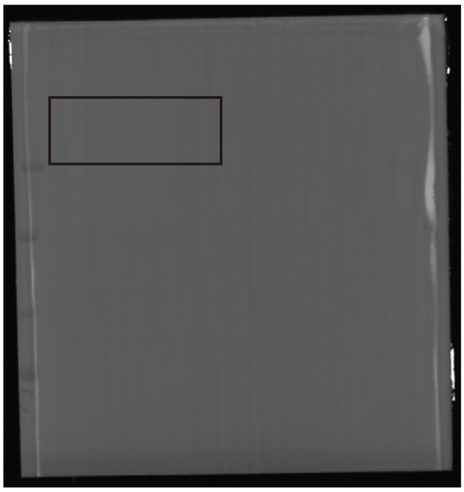

IB : GAPDH

cropped data

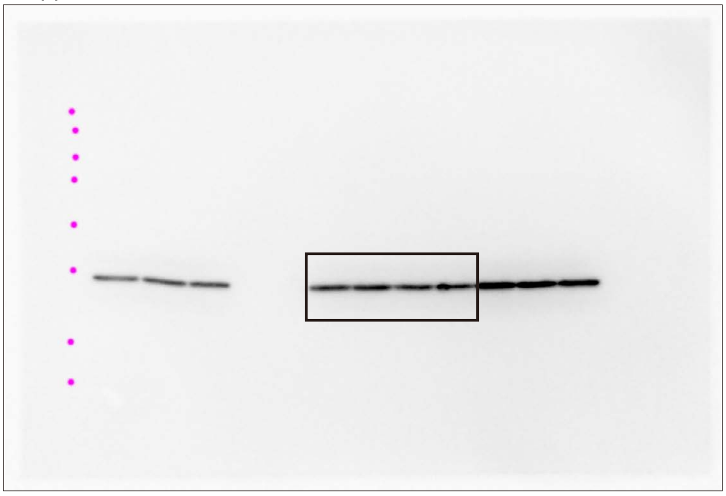

raw tiff data

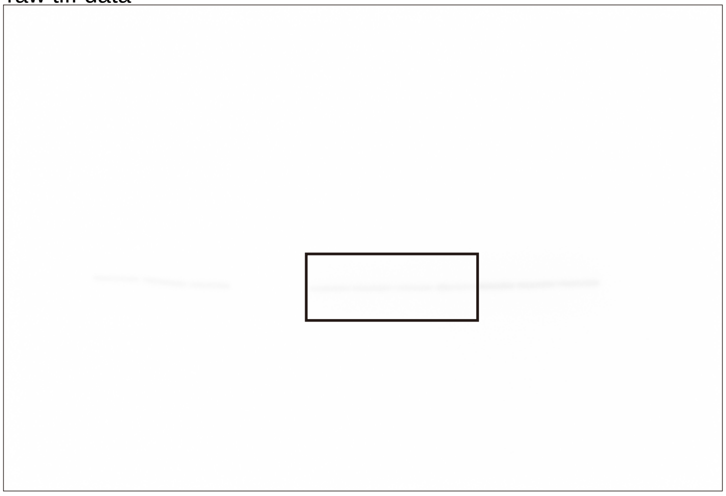

membrane

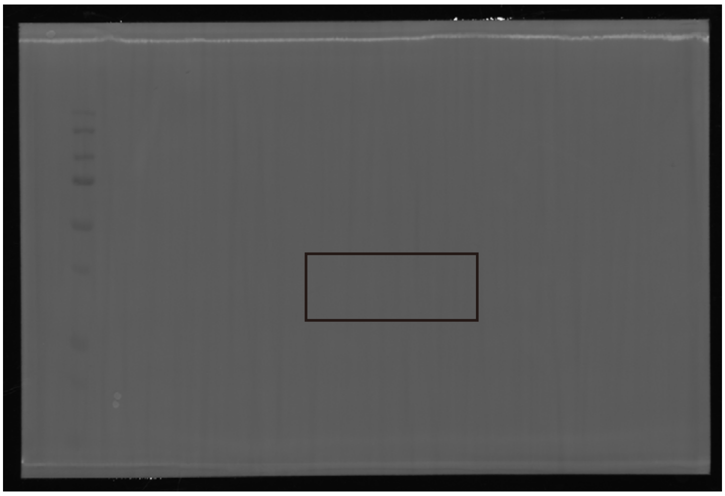

Fig. S3E

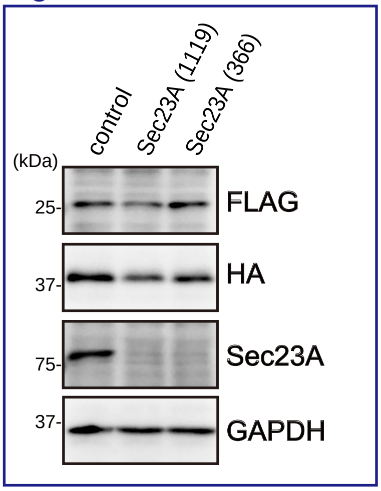

IB : FLAG

cropped data

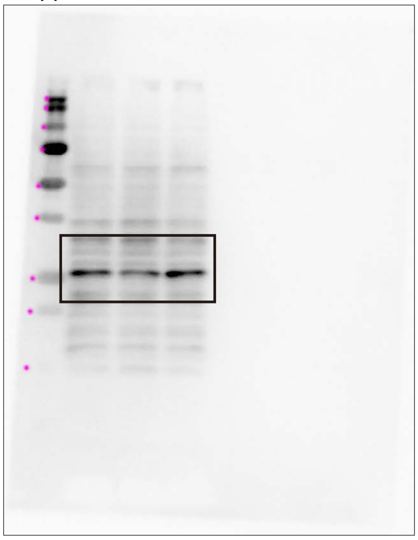

raw tiff data

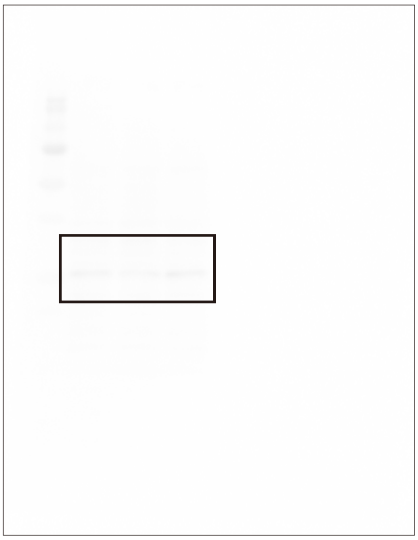

membrane

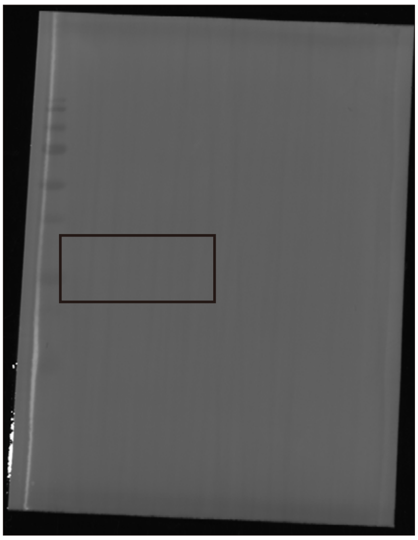

IB : HA

cropped data

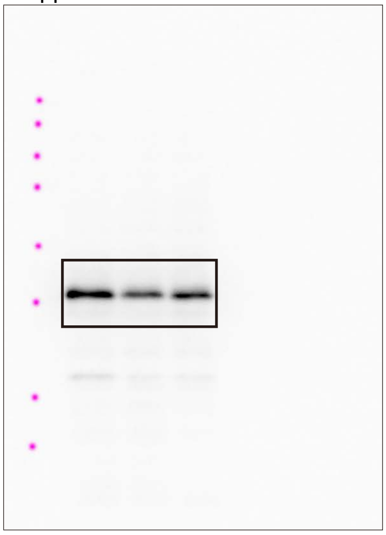

raw tiff data

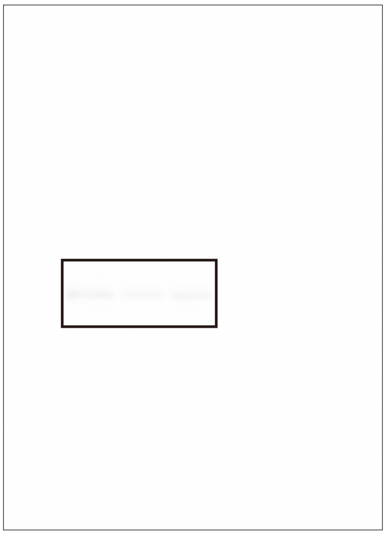

membrane

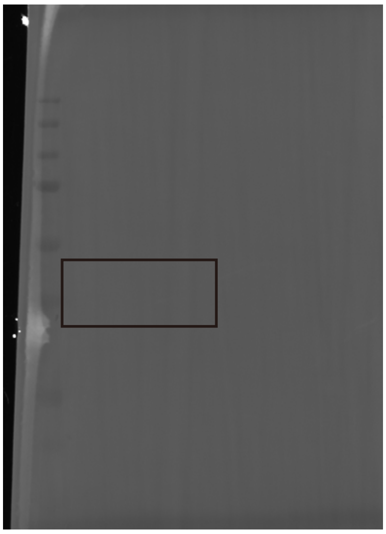

IB : Sec23A

cropped data

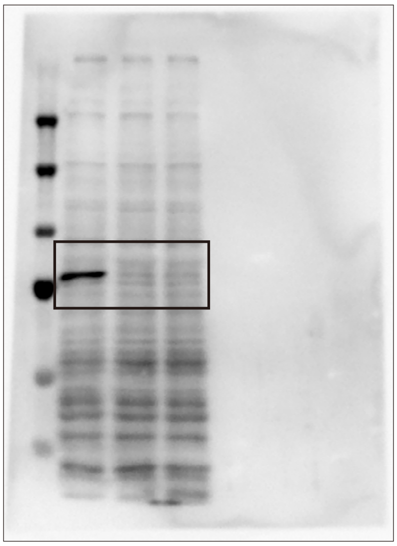

raw tiff data

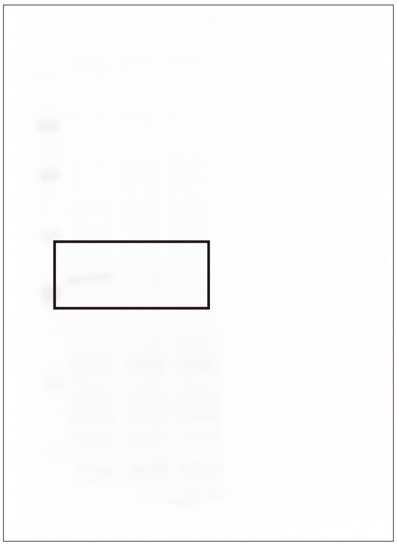

membrane

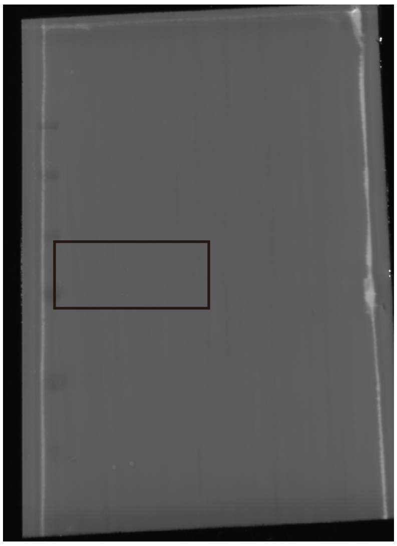

IB : GAPDH

cropped data

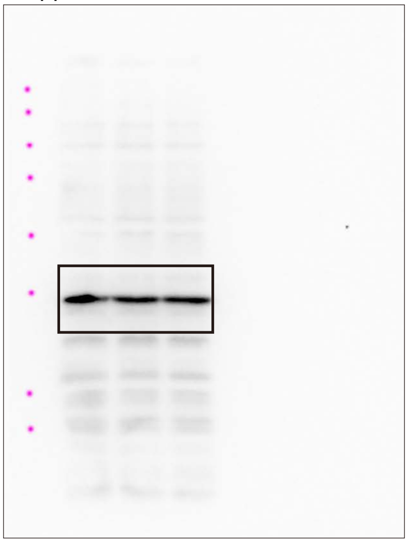

raw tiff data

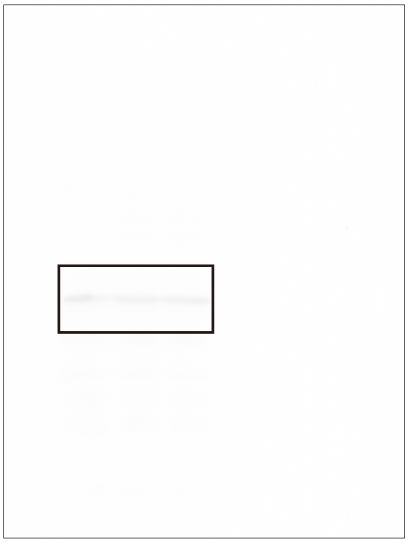

membrane

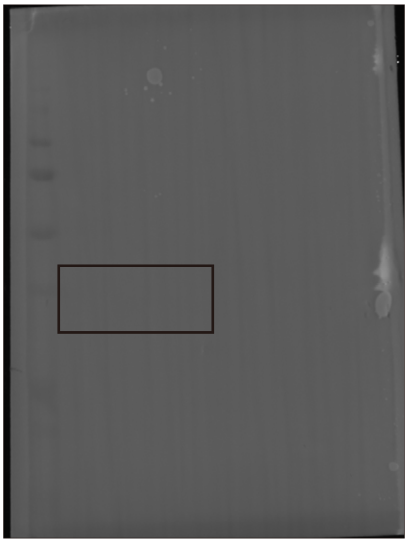

Fig. S3F

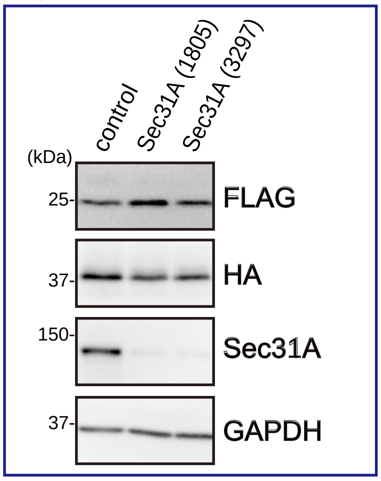

IB : FLAG

cropped data

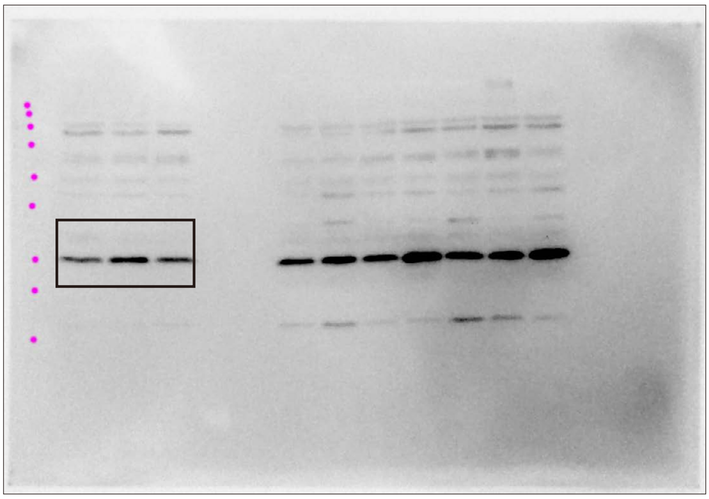

IB : HA

cropped data

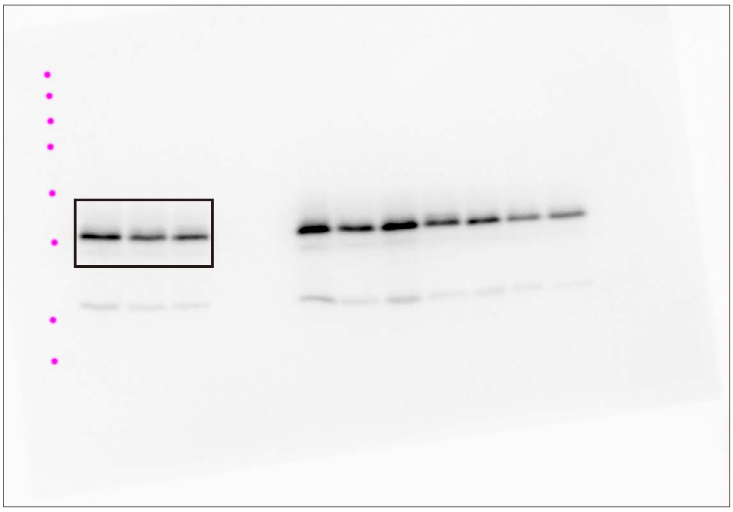

raw tiff data

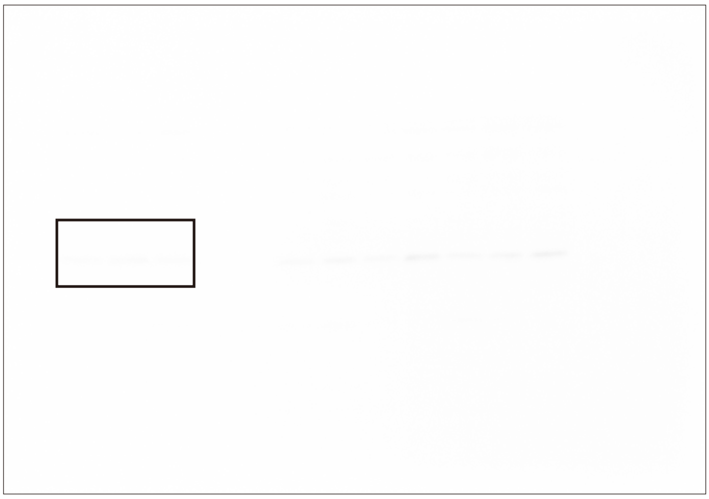

raw tiff data

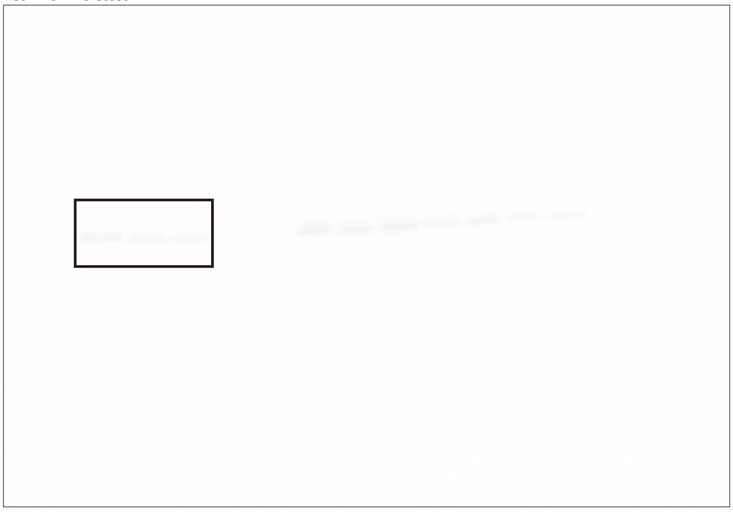

membrane

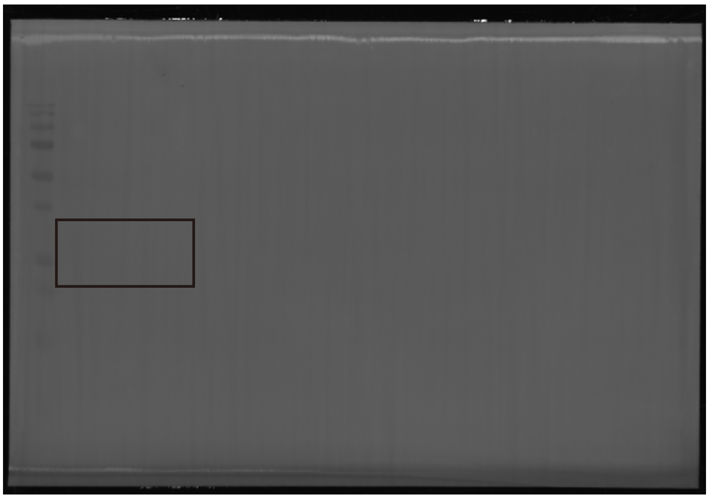

membrane

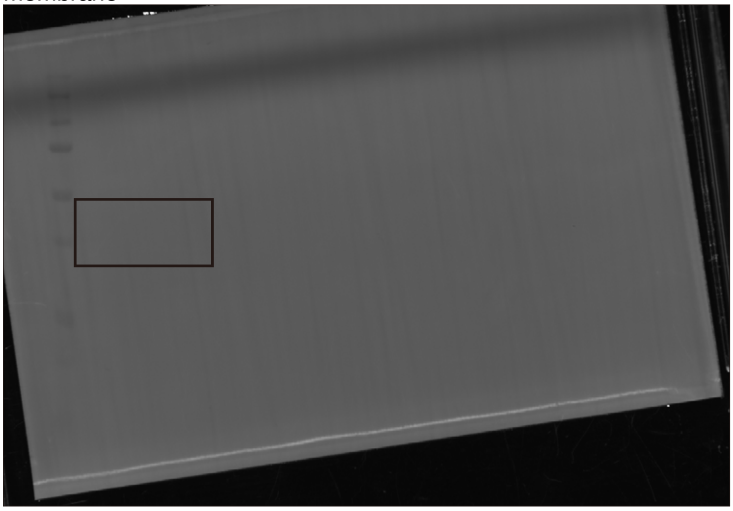

IB : Sec31A

cropped data

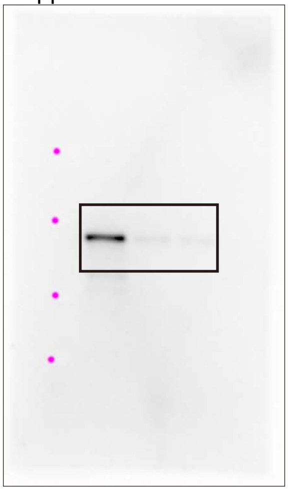

raw tiff data

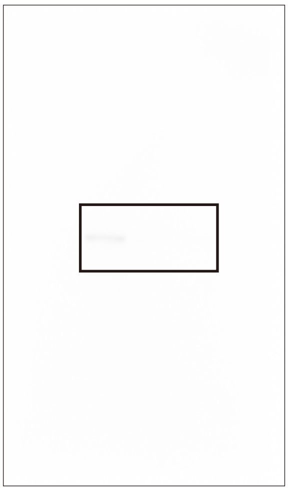

membrane

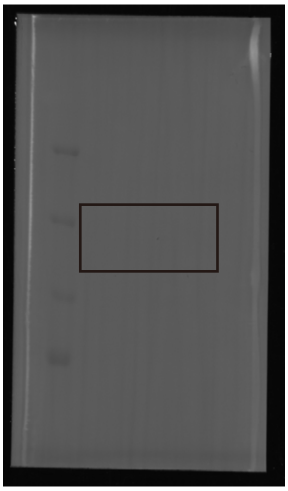

IB : GAPDH

cropped data

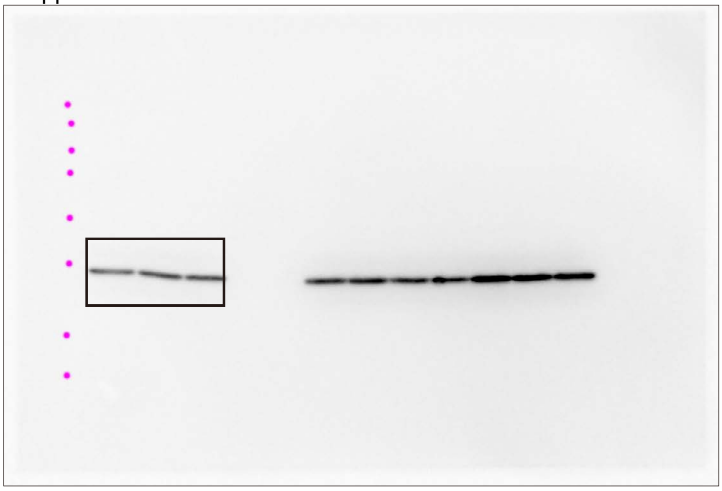

raw tiff data

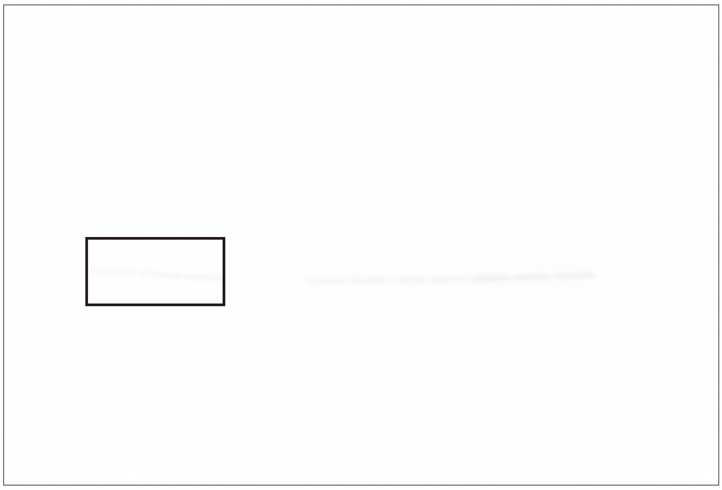

membrane

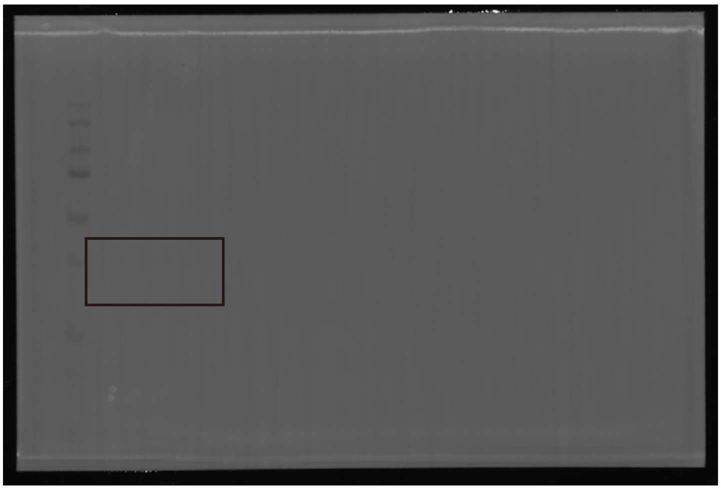

Fig. S3G

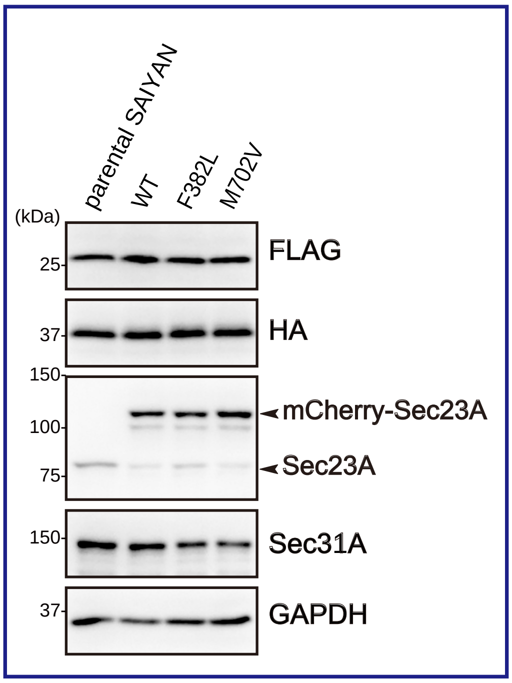

IB : FLAG

cropped data

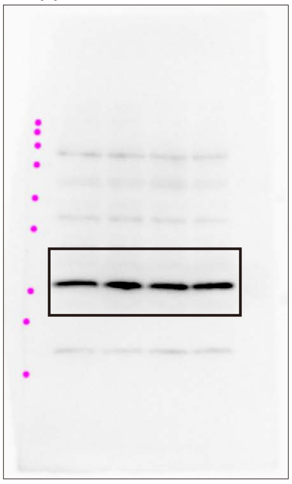

raw tiff data

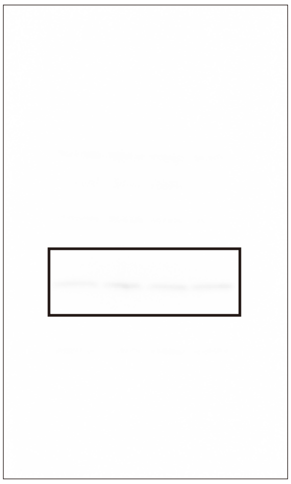

membrane

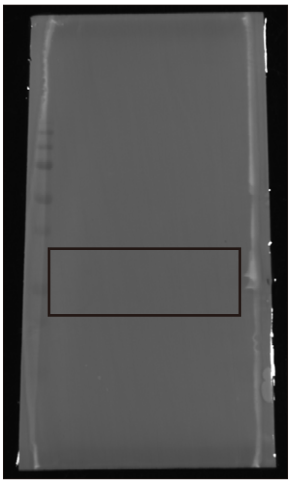

IB : HA

cropped data

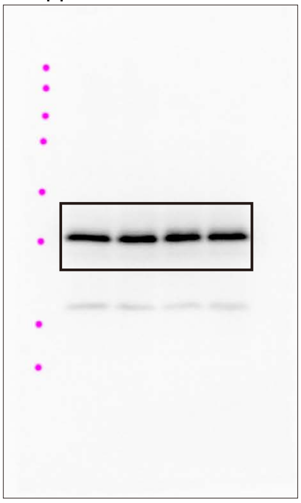

raw tiff data

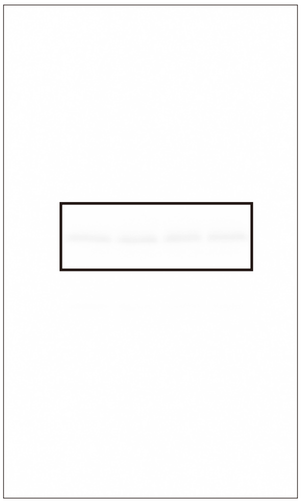

membrane

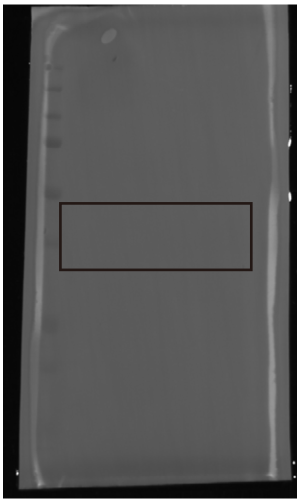

IB : Sec23A

cropped data

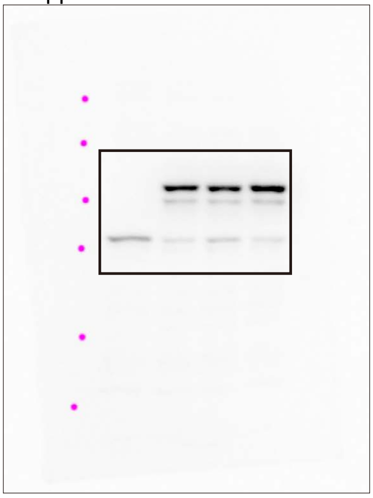

raw tiff data

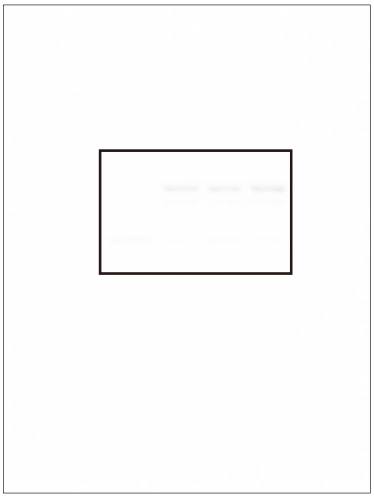

membrane

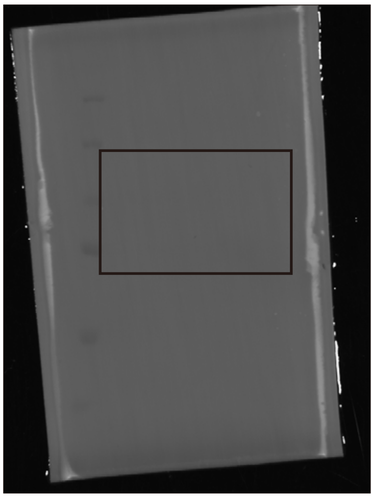

IB : Sec31A

cropped data

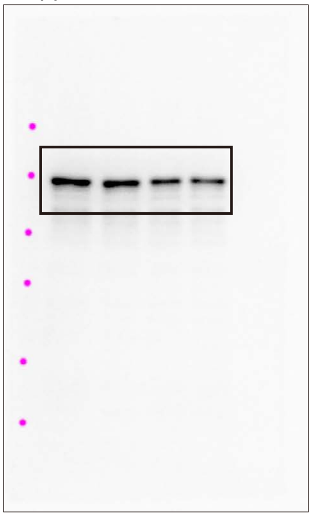

raw tiff data

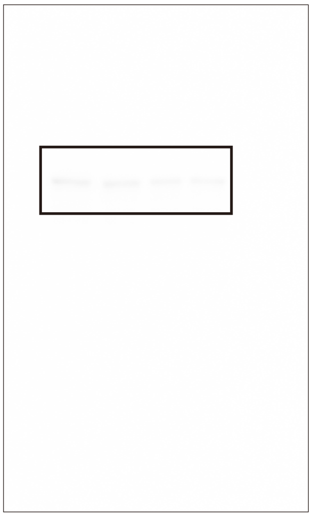

membrane

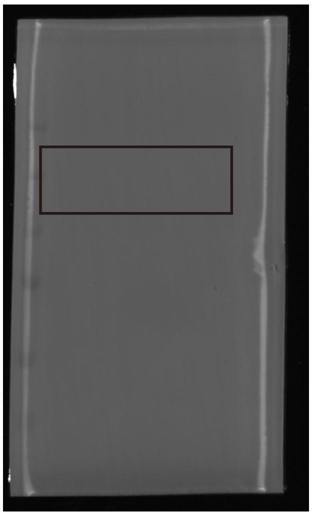

IB : GAPDH

cropped data

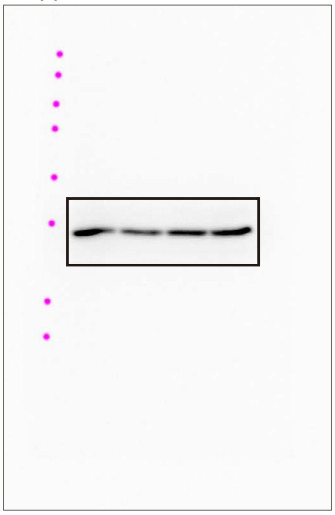

raw tiff data

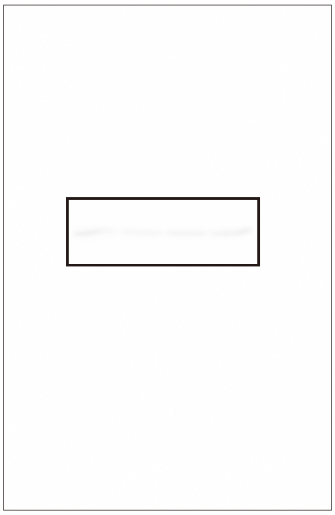

membrane

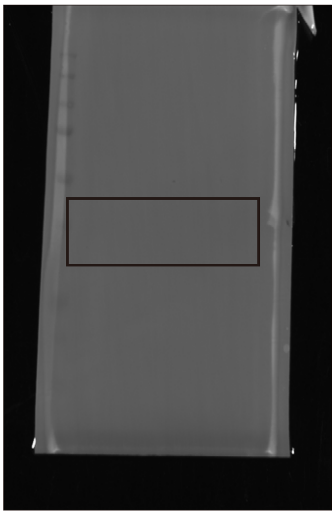

Supplement: SourceData FS3 — is the source file for Fig. S3. [file JCB_202403179_SourceDataFS3.pdf]
